# Genome-Wide Divergence in the West-African Malaria Vector *Anopheles melas*

**DOI:** 10.1534/g3.116.031906

**Published:** 2016-07-27

**Authors:** Kevin C. Deitz, Giridhar A. Athrey, Musa Jawara, Hans J. Overgaard, Abrahan Matias, Michel A. Slotman

**Affiliations:** * Department of Entomology, Texas A&M University, College Station, Texas 77843; † Department of Poultry Science, Texas A&M University, College Station, Texas 77843; ‡ Medical Research Council Unit, Banjul, Fajara, The Gambia; § Department of Mathematical Sciences and Technology, Norwegian University of Life Sciences, Ås, Norway; ** Medical Care Development International, Malabo, Equatorial Guinea

**Keywords:** *Anopheles melas*, *Anopheles gambiae*, malaria, population genomics, Pool-seq

## Abstract

*Anopheles melas* is a member of the recently diverged *An. gambiae* species complex, a model for speciation studies, and is a locally important malaria vector along the West-African coast where it breeds in brackish water. A recent population genetic study of *An. melas* revealed species-level genetic differentiation between three population clusters. *An. melas* West extends from The Gambia to the village of Tiko, Cameroon. The other mainland cluster, *An. melas* South, extends from the southern Cameroonian village of Ipono to Angola. Bioko Island, Equatorial Guinea *An. melas* populations are genetically isolated from mainland populations. To examine how genetic differentiation between these *An. melas* forms is distributed across their genomes, we conducted a genome-wide analysis of genetic differentiation and selection using whole genome sequencing data of pooled individuals (Pool-seq) from a representative population of each cluster. The *An. melas* forms exhibit high levels of genetic differentiation throughout their genomes, including the presence of numerous fixed differences between clusters. Although the level of divergence between the clusters is on a par with that of other species within the *An. gambiae* complex, patterns of genome-wide divergence and diversity do not provide evidence for the presence of pre- and/or postmating isolating mechanisms in the form of speciation islands. These results are consistent with an allopatric divergence process with little or no introgression.

The *Anopheles gambiae* complex of African malaria mosquitoesis a model system for the study of speciation (Fontaine *et al.* 2015; Mallet *et al.* 2015; Neafsey *et al.* 2015; Nosil 2012). This is partly due to its importance to human health, but also because varying levels of reproductive isolation and introgression are found between its member species (Besansky *et al.* 1994; Davidson 1962; Fontaine *et al.* 2015; Lanzaro and Lee 2013; Marsden *et al.* 2011; Powell *et al.* 1999; Slotman *et al.* 2004, 2005a,b; Weetman *et al.* 2014), chromosomal and molecular forms occur within species (Coluzzi *et al.* 2002; della Torre *et al.* 2001; Favia *et al.* 2001; Gentile *et al.* 2001; White *et al.* 2011), and contrasting patterns of intraspecific population structure have been observed between species (Deitz *et al.* 2012; Donnelly and Townson 2000; Lehman *et al.* 2003; Loaiza *et al.* 2012). The recent evolutionary analyses of 16 *Anopheles* genomes highlighted the role of adaptive introgression in the divergence of the *An. gambiae* complex (Clarkson *et al.* 2014; Fontaine *et al.* 2015; Norris *et al.* 2015), and how biological factors involved in their capacity to vector human malaria parasites have influenced the evolution of these species (Neafsey *et al.* 2015).

Eight species have now been formerly described within the *An. gambiae* complex, including two recent additions: *An. coluzzii*, formerly *An. gambiae* M molecular form, and *An. amharicus*, formerly *An. quadriannulatus* B (Coetzee *et al.* 2013). The elevation of the *An. gambiae* M form to species rank was based on ecological divergence, assortative mating (della Torre *et al.* 2001; Simard *et al.* 2009; Tripet *et al.* 2005; Aboagye-Antwi *et al.* 2015), and genetic divergence that appears to be limited to several small regions of the genome (Turner *et al.* 2005; White *et al.* 2010). The description of *An. coluzzii* therefore broke with the tradition of describing new species in the complex based on the presence of hybrid sterility (Davidson 1962; Hunt *et al.* 1998), as hybrids between *An. gambiae* and *An. coluzzii* are fully fertile (Diabaté *et al.* 2007). Thus, the description of *An. coluzzii* is aligned more with a genotypic cluster species concept (Mallet 1995) rather than a biological species concept (Mayr 1970).

A recent study on the population structure of *An. melas* throughout its range uncovered species-level genetic divergence between three population clusters (Deitz *et al.* 2012). *An. melas* is distributed along the west coast of Africa as its larval ecology is tied to brackish water, mangrove forests, and salt marshes. Nonetheless, it is an important vector of human malaria where it is found (Bryan *et al.* 1987; Caputo *et al.* 2008), with the average number of malaria infective *An. melas* bites/person/year sometimes reaching 130 (Overgaard *et al.* 2012). Coluzzi *et al.* (2002) found that some chromosomal inversions were nonrandomly distributed between *An. melas* populations, suggesting the presence of some reproductive barriers. Deitz *et al.* (2012) showed that *An. melas* is in fact divided into three genetic clusters that appear to be mostly isolated from each other. Two of these clusters are distributed on the African mainland: *An. melas* West ranges from The Gambia to Northwest Cameroon, and *An. melas* South ranges from Southeast Cameroon to Angola. A third cluster, *An. melas* Bioko, is limited to Bioko Island, Equatorial Guinea, located approximately 40 km off the Cameroonian coast (Figure 1).

**Figure 1:**
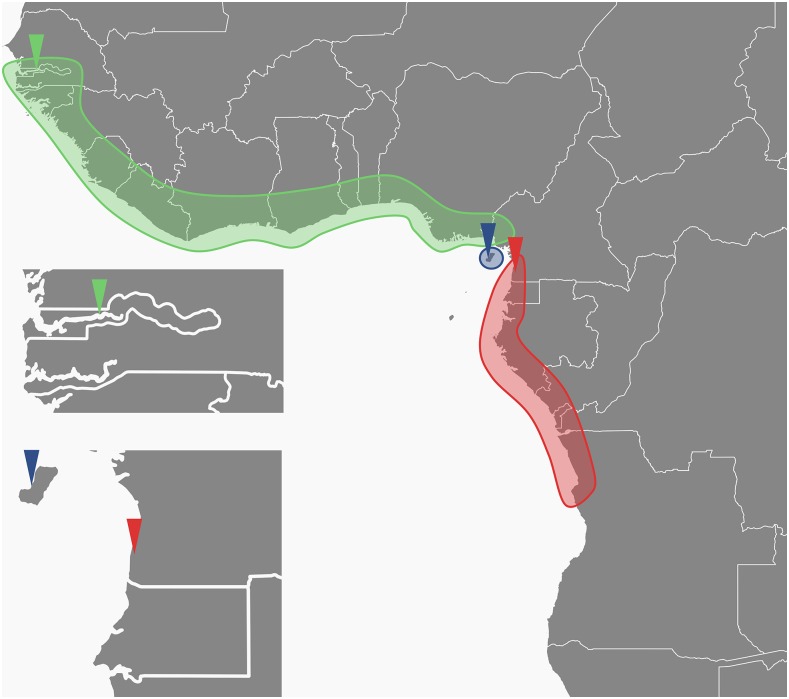
This map of West Africa illustrates the distributions of *An. melas* genetic clusters. Ranges of *An. melas* West (green), South (red), and Bioko (blue) are shown as shaded regions. Triangles show the sample locations of *An. melas* populations used to represent each *An. melas* genetic cluster. The top inset shows the collection location of Ballingho, The Gambia (green triangle, *An. melas* West), and the bottom inset shows the collection locations of Arena Blanca, Bioko Island, Equatorial Guinea (blue triangle, *An. melas* Bioko) and Ipono, Cameroon (red triangle, *An. melas* South).

No mtDNA haplotypes are shared between *An. melas* clusters, and microsatellite data indicates almost complete genetic isolation, with the exception of limited introgression into *An. melas* West from the South and Bioko, which was identified through a Bayesian analysis of population structure. Additionally, the level of genetic divergence (*F_ST_*) between *An. melas* West and South equaled or exceeded levels previously observed between *An. gambiae* and *An. arabiensis* (Slotman *et al.* 2005a; Fontaine *et al.* 2015). Interestingly, *An. melas* West and South populations are only separated by approximately 190 km of unsampled terrain along the Cameroonian coast. The high level of isolation of the *An. melas* Bioko Island population is also remarkable given the short distance to the mainland, and the very low level of genetic differentiation between Bioko Island and mainland populations of both *An. gambiae* and *An. coluzzii* (Moreno *et al.* 2007; Deitz *et al.* 2012).

An analysis of the demographic history of *An. melas* populations using approximate Bayesian computation analysis indicated that a larger ancestral *An. melas* population split into two mainland clusters through a vicariance event sometime during the last several hundred thousand years. Similarly, *An. melas* Bioko was once connected to *An. melas* West populations, but became isolated around 90,000 years before the present day, presumably due to rising sea levels (Deitz *et al.* 2012).

In the present study, we used a whole-genome, pooled-population sequencing (Pool-seq) approach (Schlötterer *et al.* 2014) to examine genome-wide patterns of diversity within, and divergence between, a representative population sample of *An. melas* West, South, and Bioko. Such an analysis may reveal whether the geographically isolated forms of *An. melas* harbor any genetically highly diverged regions of the genomes, similar to those that have been tied to premating isolation between *An. gambiae s.s*. and *An. coluzzii* (Aboagye-Antwi *et al.* 2015). The genome-wide single nucleotide polymorphism (SNP) data show that *An. melas* population clusters have high levels of genome-wide genetic differentiation, as evidenced by numerous high-*F_ST_* and fixed SNPs in each population comparison. Genetic differentiation is particularly high on the *X* chromosome, which also carries the largest number of fixed differences. Additionally, we identified candidate regions under positive selection within each *An. melas* population cluster. A lack of narrow, highly differentiated genomic regions is consistent with allopatric divergence with little or no introgression.

## Materials and Methods

### Population genomic analysis

Pool-seq was performed on DNA of *Anopheles melas* females collected from Ballingho, The Gambia (*N* = 20), Ipono, Cameroon (*N* = 23), and Arena Blanca, Bioko Island, Equatorial Guinea (*N* = 20). These populations fall within *An. melas* West, South, and Bioko Island genetic clusters, respectively (Figure 1) (Deitz *et al.* 2012). Populations were chosen based upon the high quality of DNA available to create pooled libraries for sequencing, and the lack of gene flow observed between them and neighboring *An. melas* clusters (Deitz *et al.* 2012). Mosquito collection and DNA extraction methods are as described in Deitz *et al.* (2012). We pooled equal amounts of DNA from each individual, and sequencing libraries were constructed from 1.0 µg of pooled DNA. Covaris shearing (Fisher *et al.* 2011) was used to produce approximately 200 bp inserts for each library. Libraries were bar-coded, combined, and paired-end sequenced on a single lane of the Illumina HiSequation 2000 DNA sequencing platform.

Sequencing reads were trimmed to a minimum Phred quality score of 20 and a minimum length of 50 base pairs using Trimmomatic version 0.35 (Bolger *et al.* 2014), and then mapped to the *An. gambiae* PEST P4.3 genome assembly (Holt *et al.* 2002) using Stampy (Lunther and Goodson 2011) with a substitution rate = 0.02. Stampy is designed to map DNA sequencing reads to a divergent reference genome and has been previously used for this purpose in the *An. gambiae* species complex (Smith *et al.* 2015). Sequencing reads were mapped to the *An. gambiae* genome rather than the *An. melas* genome (Neafsey *et al.* 2015) because the former is assembled into chromosomes and at the present time the *An. melas* genome is comprised of 20,229 scaffolds (Giraldo-Calderón *et al.* 2015; Neafsey *et al.* 2015). No coordinate lift-over file is available to convert the coordinates of the *An. melas* scaffolds to those of the *An. gambiae* P4.3 chromosomes. As such, we aligned our data to the *An. gambiae* genome because it allowed us to interpret population genetic statistics in the context of chromosomal location. SAM alignment files were sorted, converted to BAM format, filtered to a minimum mapping quality value (MAPQ) of 20, and converted to pileup files using SAMtools version 0.1.19 (Li *et al.* 2009).

Pileup files were used to calculate nucleotide diversity (π, Nei and Li 1979) and Tajima’s *D* (Tajima 1989) using the PoPoolation package (Kofler *et al.* 2011a). Both statistics were calculated using 100 kb, nonoverlapping sliding-windows using a minimum sequence coverage of four reads and maximum coverage of 40. We required a minimum of two reads for each allele at a polymorphic site to retain the site for further analysis. The highly repetitive nature of heterochromatic genomic regions leads to inaccurate read mapping, which biases population genetic statistics. Heterochromatic regions of the *An. gambiae* reference genome (Sharakhova *et al.* 2010) were removed for the calculation of π, Tajima’s *D*, and *F_ST_* summary statistics. Vertical gray bars in Figure 3 and Figure 4 highlight heterochromatic regions.

Multiple pileup files were created with SAMtools version 0.1.19 (Li *et al.* 2009) and transformed into synchronized pileup files using PoPoolation2 (Kofler *et al.* 2011b). This program was then used to calculate pair-wise *F_ST_* values for each SNP, and for 100 kb, nonoverlapping sliding-windows using a minimum sequencing depth of 30 × and a maximum equal to the top 2% of the sequencing depth distribution of each pool. Reads exceeding the top 2% sequencing depth threshold were excluded from our analysis to reduce the effect of sequencing and mapping bias.

We chose 30 × coverage to measure SNP and window-based *F_ST_* because it allows us to have enough coverage in both populations in a comparison to provide a genome-wide distribution of informative loci for population genomic analysis, and have enough the power to detect significant differentiation. In our initial *F_ST_* null distribution simulations, we found that coverage below this value incorporates a high level of variation in the allele frequency and *F_ST_* estimates at a single locus. Thus, a high coverage threshold allows us to be confident that differences in read coverage between populations in a comparison is not biasing our *F_ST_* calculation. We used a lower threshold for π and Tajima’s *D* (above) because these values are averaged over a 100 kb window and inaccuracy in estimates for individual loci should cancel out within each window and not introduce bias.

If significant SNPs fell within the bottom 5% of the Tajima’s *D* distribution in both populations in a pair-wise comparison (*e.g.*, *An. melas* West and South), the SNP was subjected to gene ontology analyses. These analyses excluded SNPs and low Tajima’s *D* regions that fell inside regions of heterochromatin in the *An. gambiae* reference genome. SNPs were compared to the *An. gambiae* AgamP4.4 gene set (Holt *et al.* 2002; Sharakhova *et al.* 2007) to determine if they fell within a known gene exon. The molecular function, biological process, and protein class of these genes was determined using the Panther Classification System (Thomas *et al.* 2003; Mi *et al.* 2010).

To identify regions of introgression between *An. melas* forms, we calculated Patterson’s *D*-statistic, *i.e.*, the ABBA/BABA test (Green *et al.* 2010; Durand *et al.* 2011), using the program ANGSD (Korneliussen *et al.* 2014). We used 100 kb windows to analyze patterns of introgression between *An. melas* populations throughout the genome. The ABBA/BABA test compares biased proportions of ABBA *vs.* BABA patterns across a four species lineage to identify regions of introgression between populations P_3_ and P_1_ or P_3_ and P_2_, given the following topology: {[(P_1_, P_2_)P_3_]O}, where O signifies the outgroup. Positive values of Patterson’s *D*-statistic indicate biased proportions of ABBA patterns, indicating introgression between P_3_ and P_2_, whereas negative Patterson’s *D*-statistic values indicate a biased proportion of BABA patterns, and introgression between species P_3_ and P_1_. It is important to note that this test cannot determine the direction of introgression (*i.e.*, from P_3_ to P_1_, or P_1_ to P_3_).

Patterson’s *D*-statistic was calculated using *An. gambiae* as an outgroup and using the following tree topology: {[(West, Bioko) South] *An. gambiae*}. This tree topology is strongly supported by an approximate Bayesian computation analysis of the demographic history of *An. melas* populations based upon microsatellite data (posterior probability = 0.97) (Deitz *et al.* 2012). This tree topology allowed us to test which scenario is more likely, introgression between *An. melas* South and Bioko (P_3_ and P_2_) or between *An. melas* South and West (P_3_ and P_1_). ABBA/BABA sites were included in this analysis if sequence reads had a minimum map quality score of 30, and the SNP had a minimum base quality score of 30. The ANGSD implementation of the ABBA/BABA test uses one allele sampled from each population. While this could result in a loss of power when implemented using Pool-seq data, it will not bias the number of ABBA *vs.* BABA sites (R. Nielsen, personal communication). A delete-m jackknife approach (Busing *et al.* 1999) was used to determine the standard error of the mean Patterson’s *D*-statistic on each chromosome arm, and the entire genome. We calculated a Z-score to test if ABBA or BABA counts on each chromosome arm differed significantly from the null hypothesis of Patterson’s *D*-statistic = 0 (no excess of ABBA or BABA sites), indicating introgression between two of the populations.

### Generation of an F_ST_ null distribution and false discovery rate

Previous studies using Pool-seq identified divergent genomic regions by visually inspecting sliding-window *F_ST_* graphs for high peaks (*e.g.*, Karlsen *et al.* 2013), or considered SNPs to be significant if they were four standard deviations above the mean value of the *Z*-transformed *F_ST_* distribution (*e.g.*, Montague *et al.* 2014). Others considered SNPs to be significantly differentiated between populations if their pair-wise *F_ST_* values fell in the top 0.5% of the *F_ST_* distribution, and had a Bonferroni-corrected *p*-value lower than 0.05 when subjected to a Fisher’s exact test (Kofler *et al.* 2011b; Fabian *et al.* 2012). While conservative approaches such as a Bonferroni correction reduce type I error, they may exclude a large number of biologically significant SNPs from downstream analyses (Darum 2006). Additionally, relying on the Fisher’s exact test implemented in PoPoolation2 for detecting significant differences in allele frequencies does not take into account pool size, which can influence allele frequency estimates. Thus, it only works well for studies in which pool size is considerably larger than sequencing coverage and can be ignored. In cases of small pool size, it will lead to a potentially large number of false positive results.

**Figure 2:**
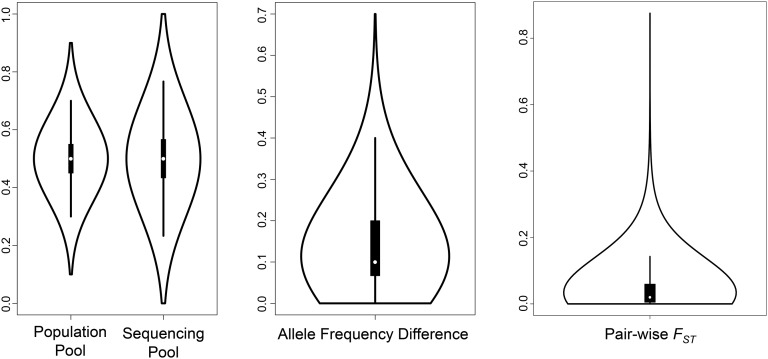
Summary violin plots of the *F_ST_* null distribution and false discovery rate simulation. The left plots show the allele frequency distribution of population and sequencing pools. The middle plot represents the difference between two randomly sampled allele frequencies drawn from the sequencing pool. The right plot shows the distribution of *F_ST_* values calculated from the distribution of allele frequency differences.

Therefore, we created a *F_ST_* null distribution by simulating *F_ST_* values observed between two samples drawn from a single population, given our pool size and sequence coverage. This null distribution allows us to determine which SNPs are significantly differentiated in our data. We created this null distribution by performing simulations in R (https://www.r-project.org). First, we drew 40 alleles (*N* = 20) from a population of 1000 individuals with a single SNP at an allele frequency of 0.5. We used an initial allele frequency of 0.5 because this value results in the largest variance of the estimated allele frequency. This step was repeated 10 million times to create our “population pool” allele distribution (Figure 2). This step simulates the pooling of individuals. We then drew 30 alleles (the minimum sequencing coverage (30 ×) used for SNP-wise and window-based *F_ST_* estimation) from our population pool allele distribution. This step was repeated 10 million times to create the “sequencing pool” allele distribution (Figure 2). This step simulates the random generation of sequencing reads from the Pool-seq DNA library. The simulation of these two sampling steps combined provides the distribution of possible allele frequency estimates.

To obtain the *F_ST_* null distribution, we drew two allele frequency values from this allele frequency distribution 10 million times and calculated the allele frequency difference between them (Figure 2). We calculated the *F_ST_* value for each of these pairs using *F_ST_ =* (*H_T −_ H_S_*) / (*H_T_*), where *H_T_* is the total population heterozygosity and *H_S_* is the subpopulation heterozygosity. This process was also repeated 10 million times to create the “pair-wise *F_ST_*” distribution. This *F_ST_* null distribution was used to find the *F_ST_* value for which the false discovery rate (FDR) ≤ 0.05. For each pair-wise population comparison, this was done by finding the threshold *F_ST_*-value for which: (*p*-value × Total SNP number) / (significant SNP number) = 0.05. Here, the “p-value” is the proportion of *F_ST_* values above the threshold *F_ST_* value in the null distribution, “total SNP number” is the number of SNPs in the population data set, and “significant SNP number” is the number of SNPs in the population data set with an *F_ST_* value above the threshold. In other words, the numerator is the expected number of false positives, and the denominator is the number of significantly differentiated SNPs in the data set.

### Data availability

The authors state that all data necessary for confirming the conclusions presented in the article are represented fully within the article. Accession numbers for raw sequence reads are provided in Table S1.

## Results

### Sequence read quality control

The sequencing effort resulted in 78,025,712 paired-end reads for *An. melas* West (Ballingho, The Gambia), 52,594,743 for *An. melas* South (Ipono, Cameroon), and 56,776,632 for *An. melas* Bioko (Arena Blanca, Bioko Island, Equatorial Guinea) (Supplemental Material, Table S1). Paired-end reads were mapped to the genome only if both forward and reverse reads survived quality and length trimming (Phred ≥ 20, length ≥ 50 bp). Mapped reads with MAPQ values greater than 20, and that mapped to chromosomes *X*, 2, or 3, were retained for further analysis (West = 52.31%, South = 26.16%, and Bioko = 38.38% of original, raw reads). These reads had a mean length of 98.7–99.1 bp for each population (Table S1). However, the mean, genome-wide read coverage per base pair varied between populations (West = 34.44, South = 17.27, and Bioko = 25.41). This factor limited the number of SNPs that met our criteria of 30 × coverage for analysis of *F_ST_* between population pools.

### Nucleotide diversity and evolution

While we used lower thresholds (minimum coverage of 4 ×) for the calculation of nucleotide diversity and Tajima’s *D*, our results show that the mean reads/bp far exceed these values on all chromosome arms in all populations (Table S1). For example, the lowest observed mean reads/bp (15.63) was on chromosome arm 3L of *An. melas* South. The 4 × threshold was used to maximize the number of variable sites within a 100 kb window included in the calculation of nucleotide diversity and Tajima’s *D*. On chromosome arm 3L of *An. melas* South, on average 36.34% of a 100 kb window exceeded the minimum coverage threshold.

**Figure 3:**
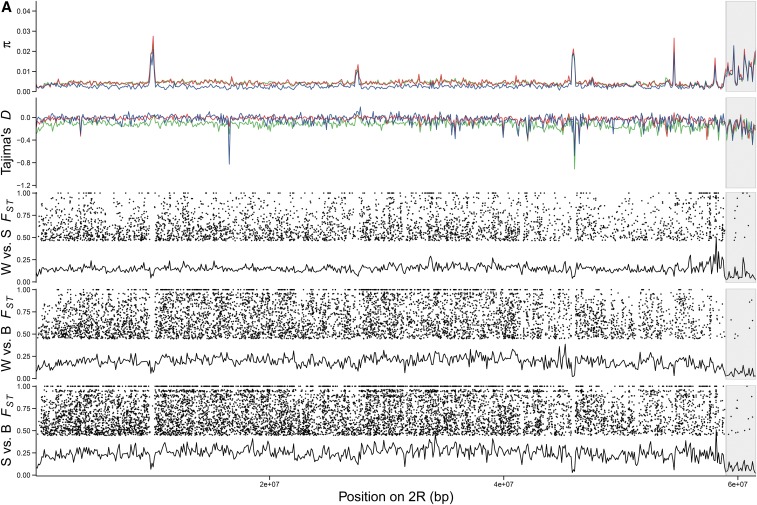

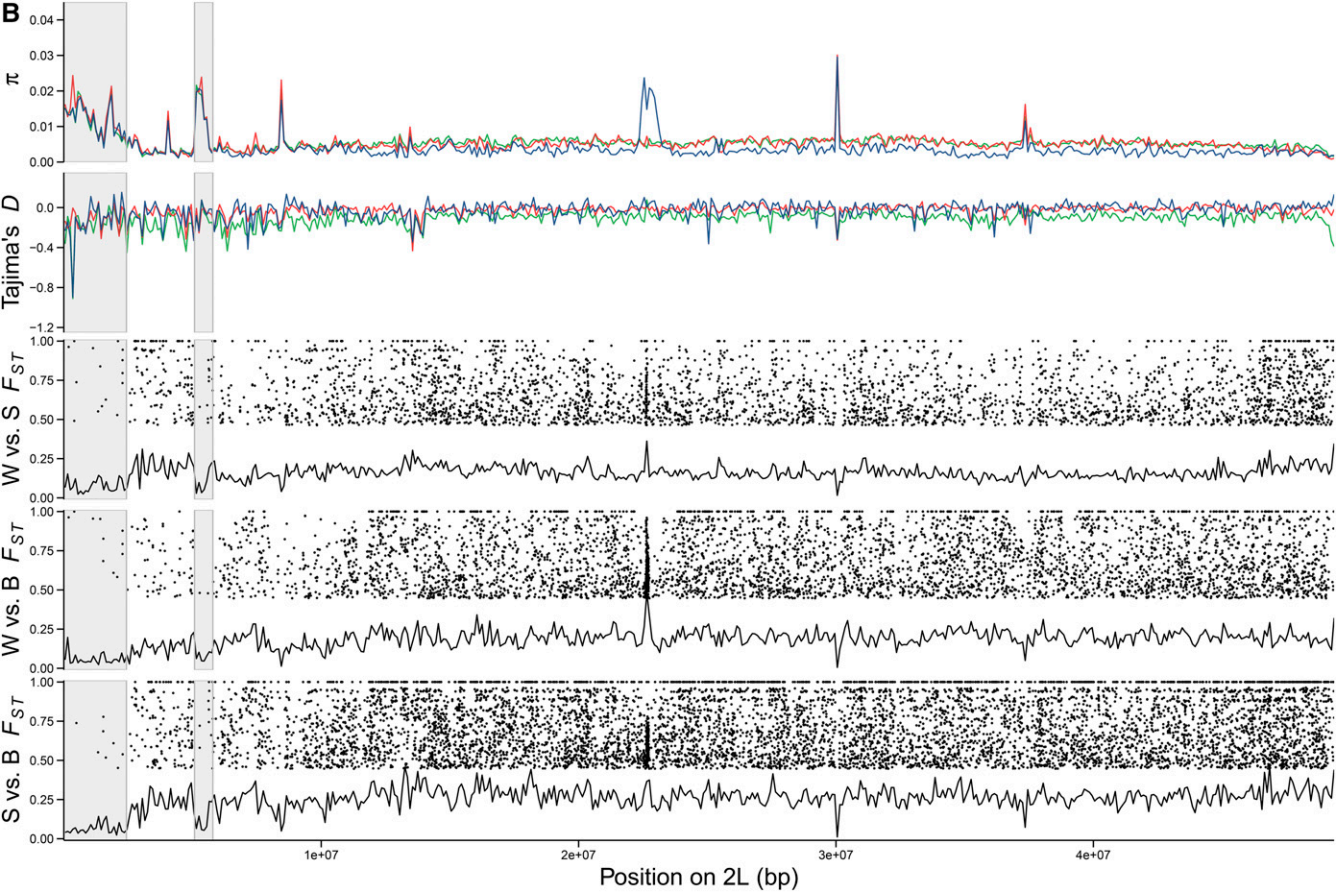

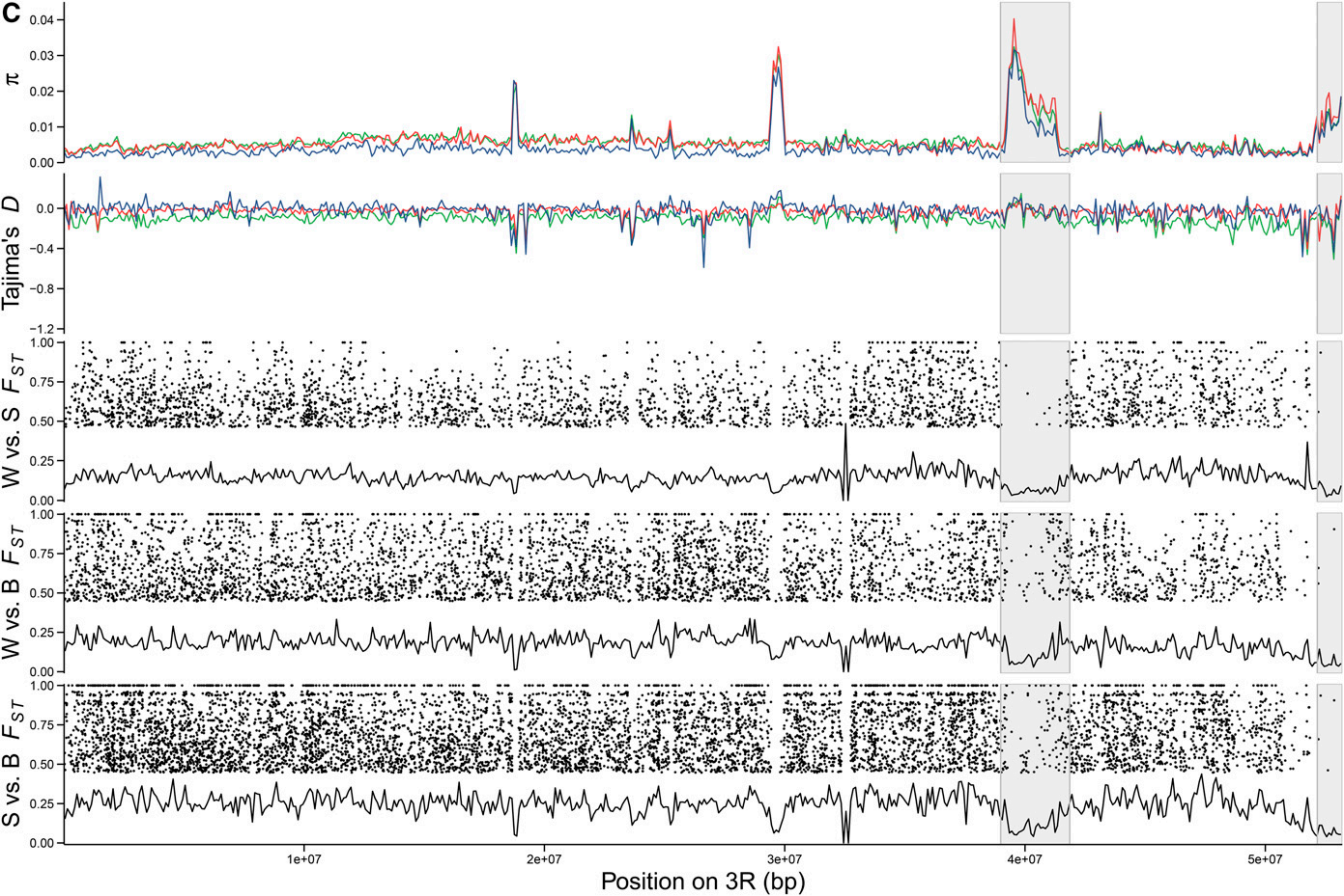

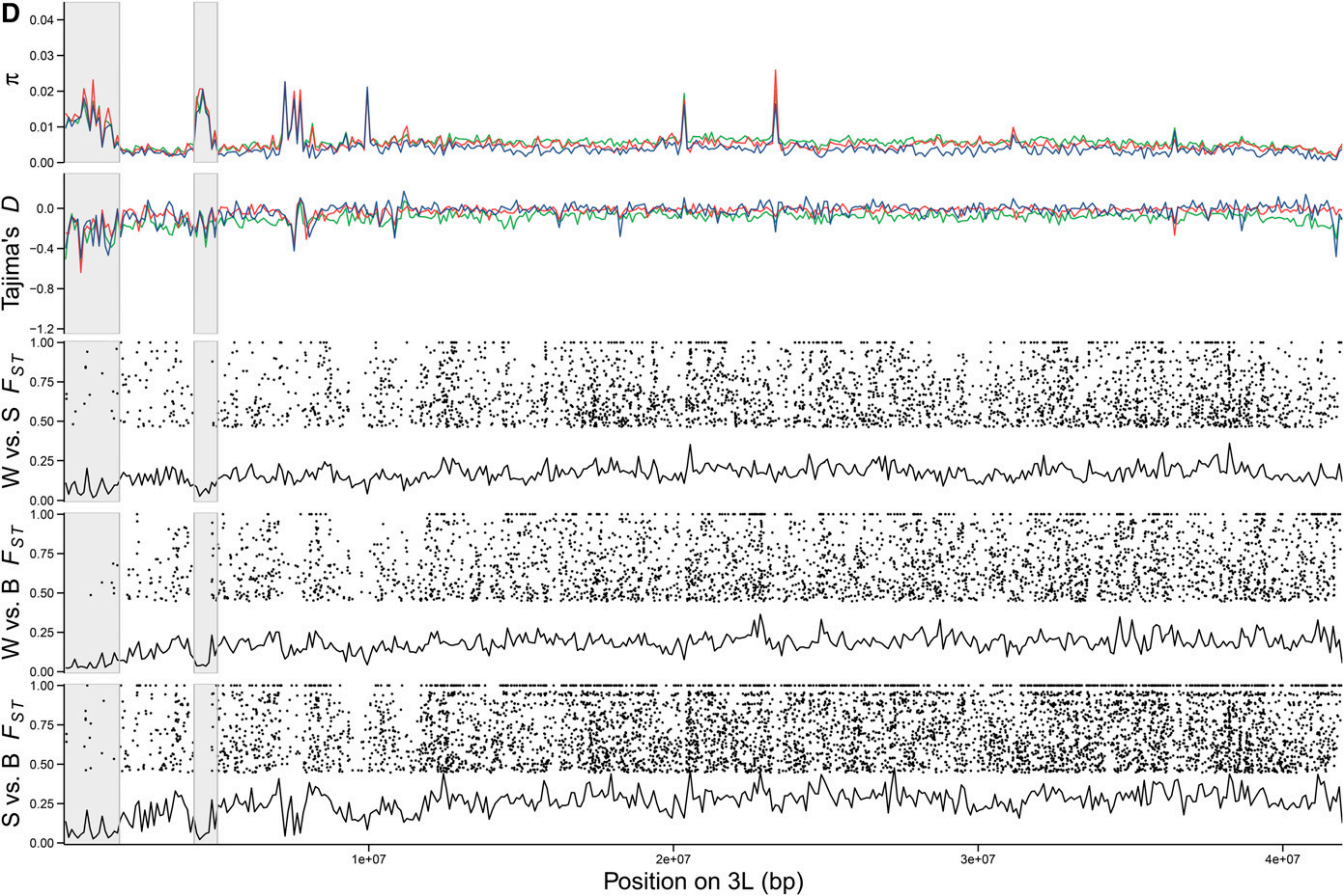

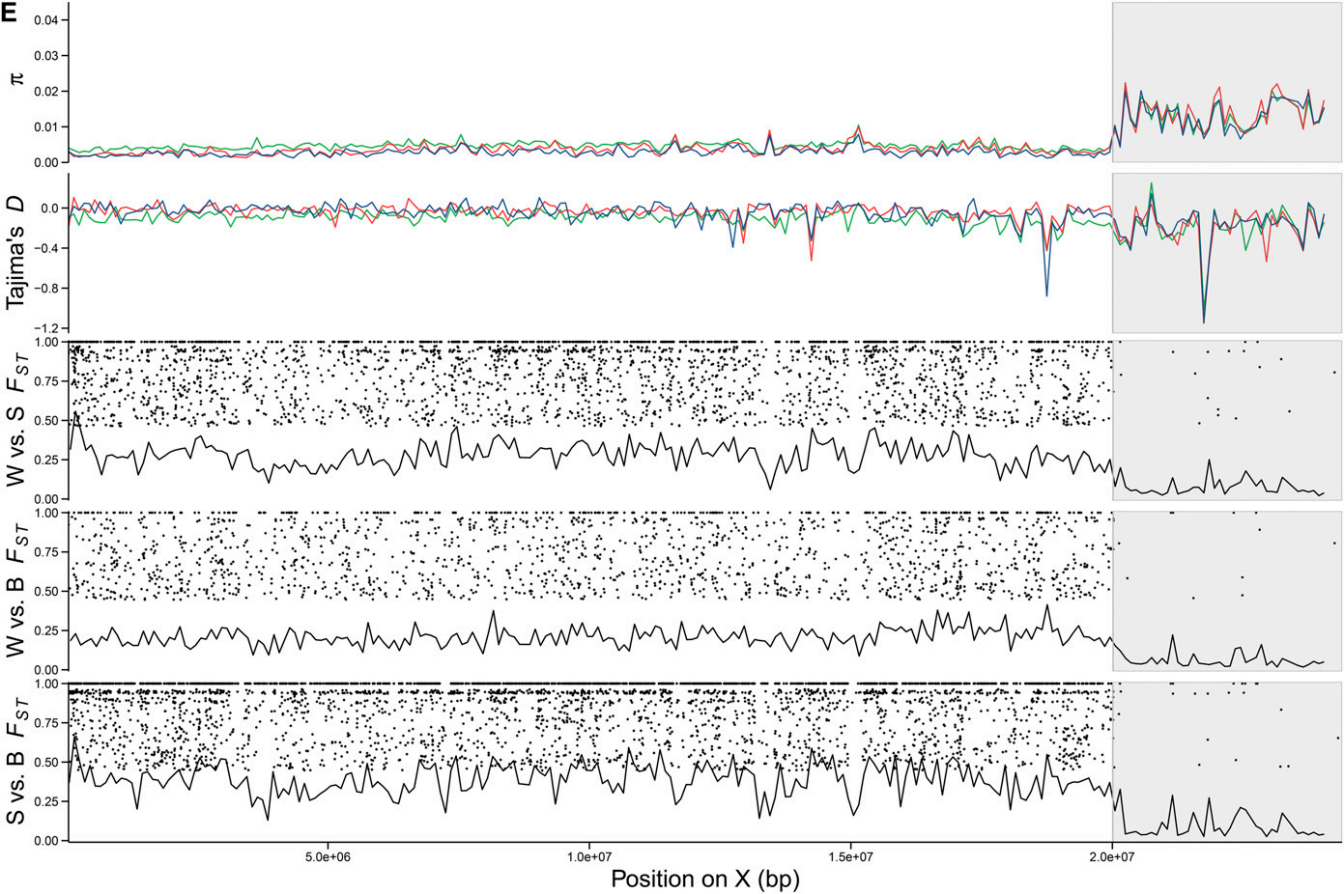
Line plots illustrate genome-wide nucleotide diversity (π) and Tajima’s *D* estimates for each chromosome arm and population based upon nonoverlapping, 100 kb sliding windows. (A–E) Green lines represent *An. melas* West, red lines represent *An. melas* South, and blue lines represent *An. melas* Bioko. *F_ST_* plots are presented for each pairwise population comparison: *An. melas* West *vs.* South (W *vs.* S), West *vs.* Bioko (W *vs.* B), and South *vs.* Bioko (S *vs.* B). The solid line indicates *F_ST_* calculated for nonoverlapping, 100 kb sliding windows, and dots indicate significant *F_ST_* SNPs. Vertical gray bars indicate regions of heterochromatin in the *An. gambiae* genome that were not included in the calculation of summary statistics.

Genome-wide nucleotide diversity across 100 kb windows was very similar in *An. melas* West from Ballingho, The Gambia (mean π = 0.0052, SEM = 4.78 × 10^−5^), and *An. melas* South from Ipono, Cameroon (mean π = 0.0048, SEM = 5.31 × 10^−5^), but perhaps not unexpectedly, was somewhat lower in *An. melas* Bioko from Arena Blanca, Bioko Island (mean π = 0.0034, SEM = 5.12 × 10^−5^, Table 1). This pattern was consistent across all chromosomes (*An. melas* West π > *An. melas* South π > *An. melas* Bioko π) (Figure 3 and Table 1). In each population, mean chromosomal nucleotide diversity was higher on the third chromosome, and lowest on *2R* or *X* (Figure 3 and Table 1). Interestingly, the patterns of nucleotide diversity are remarkably concordant between *An. melas* populations when viewed across their genomes, with the exception of a peak of high nucleotide diversity on chromosome *2L* in *An. melas* Bioko (Figure 3).

**Table 1:** Estimates of mean nucleotide diversity (π) and Tajima’s *D* for each chromosome arm and *An. melas* population, measured in 100 kb, nonoverlapping sliding windows

Population	X		2R		2L		3R		3L		Genome-Wide	
	π	Tajima’s *D*	π	Tajima’s *D*	π	Tajima’s *D*	π	Tajima’s *D*	π	Tajima’s *D*	π	Tajima’s *D*
West	0.0046	−0.100	0.0045	−0.126	0.0053	−0.108	0.0058	−0.107	0.0058	−0.093	0.0052	−0.1092
	(0.00008)	(0.0050)	(0.00009)	(0.0029)	(0.00009)	(0.0032)	(0.00013)	(0.0028)	(0.00011)	(0.0028)	(4.78 × 10^−5^)	(0.0014)
South	0.0035	−0.035	0.0045	−0.030	0.0050	−0.025	0.0053	−0.032	0.0051	−0.026	0.0048	−0.0291
	(0.00010)	(0.0054)	(0.00010)	(0.0024)	(0.00010)	(0.0026)	(0.00014)	(0.0025)	(0.00012)	(0.0025)	(5.31 × 10^−5^)	(0.0012)
Boiko	0.0029	−0.042	0.0029	−0.038	0.0035	−0.024	0.0037	−0.022	0.0039	−0.021	0.0034	−0.0287
	(0.00008)	(0.0070)	(0.00009)	(0.0037)	(0.00013)	(0.0037)	(0.00012)	(0.0039)	(0.00012)	(0.0038)	(5.12 × 10^−5^)	(0.0018)

Values in parentheses indicate the standard error of the mean for each statistic. Regions of heterochromatin in the *An. gambiae* genome were removed from summary statistics.

Tajima’s *D* was calculated to identify genomic regions that may be evolving under positive selection in each population. Mean Tajima’s *D* was negative for all populations, indicating a deviation from neutral evolution (*D* = 0) (Figure 3 and Table 1). Various low Tajima’s *D* regions are shared between all three populations, although some low Tajima’s *D* windows are unique to a single population (Figure 3). While broad patterns of Tajima’s *D* for each population are similar across their genomes, the genome-wide mean Tajima’s *D* of *An. melas* West is over three times lower than that of *An. melas* South and Bioko (Figure 3 and Table 1).

### F_ST_ null distribution

To determine significance thresholds for genetic differentiation (*F_ST_*) between the three *An. melas* populations, the null distribution of allele frequency differences was determined based on our pooling and sequencing coverage using simulations. Next, two values were randomly drawn from this distribution to calculate an *F_ST_* value. Each step of the simulation was repeated 10 million times to create each distribution. The first step in this simulation created a population pool with a mean allele frequency of 0.5 and a range of 0.1–0.9 (Figure 2 and Table 2). The second step created a sequencing pool distribution with a mean allele frequency of 0.5 and a range of 0.0–1.0. The final pair-wise *F_ST_* null distribution ranges from 0.0 to 0.875 and has a mean of 0.046 (Figure 2 and Table 2). For each *An. melas* pair-wise population comparison, the *F_ST_* value corresponding to FDR = 0.05 was determined and set as the significance threshold for the SNP-wise *F_ST_* analyses. These significance thresholds between the populations are *F_ST_* = 0.463 for West-South, *F_ST_* = 0.446 for West-Bioko, and *F_ST_* = 0.402 for South-Bioko. While these values are high due to relatively small pool sizes and low sequencing coverage, this conservative approach reduces the number of false positive results.

**Table 2:** Summary statistics of the *F_ST_* null distribution and false discovery rate simulation

Summary Statistic	Population Pool Distribution	Sequencing Pool Distribution	Allele Frequency Difference Distribution	Pairwise *F_ST_* Distribution
Minimum	0.100	0.000	0.000	0.000
Q1	0.450	0.433	0.067	0.005
Median	0.500	0.500	0.100	0.020
Mean	0.500	0.500	0.135	0.046
Q3	0.550	0.567	0.200	0.060
Maximum	0.900	1.000	0.700	0.875

### Genetic differentiation and introgression

Significant genetic differentiation between the three *An. melas* population clusters extends across the entire genome (Table 3 and Table S2), and includes fixed SNPs on all chromosome arms (Figure 3 and Table 3). Even though the Ipono, Cameroon and Arena Blanca, Bioko Island populations, which represent *An. melas* South and Bioko, respectively, are geographically close compared to the Ballingho, The Gambia (*An. melas* West), they are the most differentiated (Q1 = 0.018, median *F_ST_* = 0.033, mean *F_ST_* = 0.114, Q3 = 0.091), followed by the West and Bioko (Q1 = 0.016, median *F_ST_* =0.028, mean *F_ST_* = 0.076, Q3 = 0.055), and West and South (Q1 = 0.021, median *F_ST_* = 0.034, mean *F_ST_* = 0.075, Q3 = 0.062) (Table S2). *An. melas* South and Bioko also have the highest number of significantly differentiated (39,730, 8.56% of total) and fixed SNPs (5387, 1.16% of total) between them (total SNPs = 463,910), followed by West and Bioko [significant = 21,427 (3.81% of total), fixed = 1724 (0.31% of total), total SNPs = 562,493], and West and South [significant = 17,117 (2.76% of total), fixed = 1602 (0.26% of total), total SNPs = 621,184] (Table 3). It should be noted that the number of SNPs in each population comparison is influenced by differences in mapping coverage between the populations (Table 3 and Table S1). However, divergence between *An. melas* South and the other populations was largest, whereas this population has the lowest number of mapped reads.

**Table 3:** Number of significant and fixed SNPs per chromosome in each pair-wise *An. melas* population comparison

	X		2R		2L		3R		3L		Genome-Wide	
Comparison	Fixed	Sig.	Fixed	Sig.	Fixed	Sig.	Fixed	Sig.	Fixed	Sig.	Fixed	Sig.
West - South	879	3028	185	3853	202	3624	116	3340	220	3272	1602	17,117
West - Bioko	319	1810	439	6373	403	5061	299	4671	264	3512	1724	21,427
South - Bioko	1725	4324	981	10,396	1110	9197	692	8825	879	6988	5387	39,730

Regions of heterochromatin in the *An. gambiae* genome were removed from summary statistics. Sig., significant.

The *X* chromosome has a disproportionately large number of fixed and significant SNPs (Figure 3 and Table 3) in both West and South and South and Bioko population comparisons. This pattern of elevated *F_ST_* extends across the entire *X* chromosome (Figure 3). This could potentially be the result of increased genetic drift acting on polymorphisms due the lower effective population size of the *X* chromosome. Interestingly, however, this *X* chromosome effect is not obvious between *An. melas* West and Bioko, the two most recently diverged groups.

We performed a gene ontology analysis on genes within windows that show evidence of nonneutral evolution (low Tajima’s *D*). First we identified 100 kb sliding windows with the lowest 5% Tajima’s *D* values for each population (genome-wide, excluding heterochromatic regions) (*D* < −0.200, −0.096, and −0.148 for *An. melas* West, South, and Bioko, respectively). Next, we identified genes inside these windows that harbored SNPs with significant *F_ST_* values in each pair-wise comparison. The West-South comparison yielded 95 significant SNPs located inside the exons of 64 genes. The molecular functions of these genes are associated with binding, catalytic activity, nucleic acid binding transcription factor activity, and receptor activity, among others (Table S3). The West-Bioko comparison yielded 79 significant SNPs located inside exons of 62 genes and the South-Bioko comparison yielded 188 significant SNPs located inside exons of 127 genes (Table S3). The molecular functions associated with these genes are similar to those found in the West-South example. The most commonly found molecular functions (across all comparisons) include binding, catalytic activity, and nucleic acid binding transcription factor activity, and some genes are common among population comparisons (Table S3).

Common biological processes in all population comparisons include biological regulation, cellular processes, localization, and metabolic processes (Table S4). The South-Bioko comparison had 161 biological process gene ontology hits associated with the 127 genes in this analysis. The most frequent hits to protein classes across all comparisons were found in the hydrolase category, followed by proteases, nucleic acid binding proteins, proteases, and transcription factors (Table S5).

**Figure 4:**
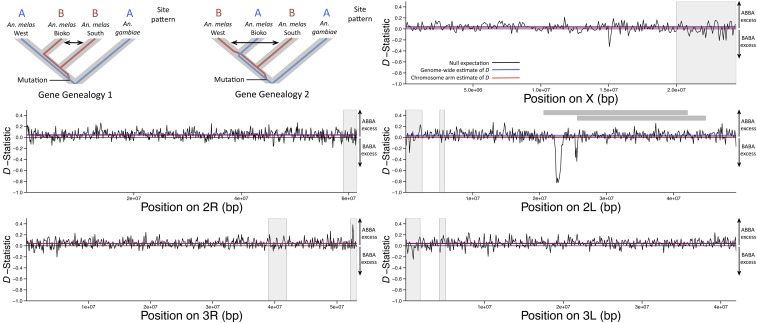
Line plots illustrate genome-wide values of Patterson’s *D*-statistic for each chromosome arm for the *An. melas* population tree {[(West,Bioko)South]*An. gambiae*}. Positive values indicate an excess of ABBA patterns and negative values indicate a biased proportion of BABA patterns. Horizontal black lines indicate the null expectation, no ABBA or BABA excess (*D* = 0). Horizontal blue lines indicate the genome-wide estimate of Patterson’s *D*, and horizontal red lines indicate the average Patterson’s *D* for each chromosome arm. Vertical gray bars indicate regions of heterochromatin in the *An. gambiae* genome that were not included in the calculation of summary statistics. Horizontal gray bars in the chromosome arm 2L panel indicate the locations of the 2La/+ (top) and 2La^2^/+ (bottom) inversions. The top left panel demonstrates the ABBA *vs.* BABA patterns in the context of the *An. melas* tree, where an ABBA pattern indicates introgression between *An. melas* Bioko and South, and a BABA pattern indicates introgression between *An. melas* West and South (arrows).

Our analysis of introgression between *An. melas* populations was based on the topology {[(West, Bioko) South] *An. gambiae*} (Deitz *et al.* 2012), and screened for introgression between *An. melas* South and Bioko or South and West. This test found a genome-wide, positive deviation of the *D*-statistic (mean *D*-statistic = 0.040, Z-score = 21.80, Table S6), indicating an excess of ABBA sites and ancient or weak introgression between *An. melas* South and Bioko. An exception to this pattern was found on chromosome 2L (∼22.25–23.45 Mb), where *D*-statistic windows with a strong, negative deviation from zero (as low as −0.83) suggest recent *An. melas* South and West introgression (Figure 4). Interestingly, this introgression block overlaps precisely with a region of high nucleotide diversity in *An. melas* Bioko (Figure 3), and falls between the proximal breakpoint of the *2La* chromosomal inversion (which is fixed for the standard arrangement *in An. melas*) and the proximal breakpoint of the *2La^2^* chromosomal inversion (which is polymorphic within *An. melas*) (Coluzzi *et al.* 2002: Sharakhov *et al.* 2006; White *et al.* 2007). The *2La^2^* inversion is specific to *An. melas* and is polymorphic within it (Coluzzi *et al.* 2002). *An. melas* collected from Guinea Bissau and Cotonou, Benin (inside the range of the *An. melas* West cluster, Figure 1) share the standard arrangement (*2L+^a2^*), while *An. melas* collected from Democratic Republic of the Congo (likely belonging to the *An. melas* South genetic cluster) are polymorphic for the standard and inverted arrangements (*2La^2^* and *2L+^a2^*) (Coluzzi *et al.* 2002).

## Discussion

Population genomic analysis of *An. melas* West, South, and Bioko Island identified significant, genome-wide genetic differentiation, including the presence of numerous fixed SNPs throughout the genome in all *An. melas* population comparisons. Previous work based on microsatellites and mtDNA markers indicated levels of differentiation between *An. melas* forms that are on a par with, or exceed, those observed between *An. gambiae* and *An. arabiensis* (Deitz *et al.* 2012). Species pairs in the *An. gambiae* complex with comparable genetic differentiation are separated by strong pre- and postmating isolation (Marchand 1983; Okereke 1980; Slotman *et al.* 2004; Weetman *et al.* 2014). Recently, the M and S molecular forms of *An. gambiae* were raised to species level (Coetzee *et al.* 2013) based on well-documented ecological and some behavioral differences. These species have diverged considerably less than the three *An. melas* genetic clusters throughout most of their genomes but have several regions of high differentiation. This is not the case for the three *An. melas* forms where, with the exception of a chromosome-wide *X* effect, genetic differentiation is distributed mostly evenly across the genome. This is consistent with a process of allopatric divergence with little gene flow/introgression. No evidence for “speciation islands”, genomic regions with high levels of divergence that are maintained in the face of extensive hybridization gene flow (Turner *et al.* 2005), was found in this study.

We used a simulation approach to construct an *F_ST_* null distribution and FDR that incorporates both pool-size and sequencing coverage. To our knowledge, this is the first time that this approach has been applied to a Pool-seq study. This allowed us to determine the *F_ST_* significance threshold for each pair-wise population comparison. In doing so, we assumed a starting allele frequency of 0.5, which results in the largest variance in the subsequent sampling steps of the simulation. In addition, we used a sequencing coverage of 30 × for our simulations, which was the minimum sequencing coverage we required for *F_ST_* calculations in our empirical analysis. Therefore, our approach is conservative. A downside of our approach is that it does not provide q-values for individual SNPs, though our method could be adapted to do so in the future.

Intrapopulation nucleotide diversity in *An. melas* revealed remarkably similar patterns of variation across the genomes of each population (Figure 3 and Table 1). This shared pattern may be attributed to shared ancestry and genome organization (*e.g.*, chromosomal inversions). Additionally, selective constraints on many genes may be similar between these populations, as the ecology may be largely shared between forms. A single peak in nucleotide diversity on chromosome *2L* of *An. melas* Bioko is the exception. Interestingly, the results of the ABBA/BABA test suggested that this exact region introgressed between *An. melas* South and West (Figure 4). This highly surprising overlap suggests to us an alternative explanation: recent introgression of this region from *An. gambiae* (or more likely, the closely related *An. coluzzii*, see below), the outgroup species in the ABBA/BABA test, into *An. melas* Bioko. This would also create a pattern of BABA excess (suggesting introgression between *An. melas* South and West) and could explain the remarkably high nucleotide diversity in Bioko Island in this particular region. Both *An. coluzzi* and *An. melas* are present on Bioko Island (Overgaard *et al.* 2012), female hybrids between the two species are fertile (Davidson 1962), and extensive introgression between various species in the complex was recently documented (Fontaine *et al.* 2015). *An. gambiae s.s*. (*i.e.*, *An. gambiae* S form) was eliminated from Bioko Island through a malaria control campaign, and only *An. coluzzii* (*i.e.*, *An. gambiae* Forest-M form) remains (Overgaard *et al.* 2012).

Genome-wide Patterson’s *D*-statistic values from the ABBA/BABA test also suggests a slight bias toward a low level of ancestral introgression between *An. melas* South and Bioko (*vs.* between West and South). This finding is perhaps not surprising considering the geographical proximity of the *An. melas* South and Bioko populations used in this study (Ipono, Cameroon and Arena Blanca, Bioko Island, Equatorial Guinea, respectively) (Figure 1) in comparison to *An. melas* from Ballingho, The Gambia, which was our representative population of *An. melas* West.

Measures of nucleotide diversity in *An. melas* populations are less than half of the mean chromosomal nucleotide diversity values observed in *An. gambiae* (S form) populations collected from the north and south of Cameroon (0.008–0.15, Cheng *et al.* 2012). This may reflect a lower *Ne* due to the patchy distribution of *An. melas* populations compared to *An. gambiae* (Athrey *et al.* 2012; Deitz *et al.* 2012). Genome-wide nucleotide diversity is the lowest in *An. melas* Bioko, which likely reflects a smaller effective population size (*N_e_*) compared to the other *An. melas* populations. Previous findings also found that the Bioko Island population harbors lower levels of rarefied allelic richness at microsatellite loci, far fewer mitochondrial DNA haplotypes, and a much lower *N_e_* compared to mainland populations (Deitz *et al.* 2012). An alternative explanation of lower diversity due to founder effects is not supported by a previous Approximate Bayesian Computation analysis of the demographic history of these populations, which indicated that all three *An. melas* forms separated through vicariance events (Deitz *et al.* 2012).

Mean chromosomal Tajima’s *D* and nucleotide diversity were lowest on the *X* chromosome for *An. melas* South and Bioko (Table 1), and nucleotide diversity of the *An*. *melas X* chromosome was the second lowest of any chromosome arm. This may be due to positive selection on (partially) recessive alleles acting more strongly on the *X* chromosome. These findings are in agreement with an effects model (SnIPRE) analysis of natural selection between *An. melas* West, South, and Bioko Island populations, which found an increased selection effect of the *An. melas X* chromosome (Struchiner *et al.*, unpublished results). Low diversity on the *X* chromosome of *An. melas* populations is consistent with findings in *An. gambiae s.s*. (Cohuet *et al.* 2008; Holt *et al.* 2002; Wilding *et al.* 2009) and *An. arabiensis* (Marsden *et al.* 2014). Introgression between member species of the *An. gambiae* complex is well documented (Fontaine *et al.* 2015), but is limited between the *X* chromosome of *An. gambiae s.s*. and other members of the complex due to the *Xag* inversion, which covers ∼60% of the *An. gambiae* s.s. *X* chromosome. The *Xag* inversion suppresses recombination between the *An. gambiae* and *An. arabiensis X* chromosomes, and plays a large role in their postzygotic reproductive isolation (Slotman *et al.* 2004, 2005b), preventing introgression. This suppressed introgression of the *X* chromosome between *An. gambiae* and *An. arabiensis* may have contributed to reduced nucleotide diversity on the *X* in these species (Marsden *et al.* 2014). Reduced introgression of the *X* chromosome may also contribute to its lower nucleotide diversity in *An. melas*, although its lower effective population size resulting in higher levels of genetic drift is probably a more important factor.

Mean Tajima’s *D* was over three times lower in *An. melas* West as compared to the South and Bioko. As this is a genome-wide effect, it likely is the result of demographic factors, such as a recent population bottleneck in the *An. melas* West population analyzed. Windows of low Tajima’s *D* are found throughout the genomes of the *An. melas* populations, which may indicate that these regions harbor genes under positive selection. Notably, very similar patterns of genome-wide Tajima’s *D* are found in each *An. melas* population cluster. This suggests that while geographic isolation of *An. melas* clusters has greatly reduced gene flow between them, their resulting genetic differentiation is likely not a result of diverging selection pressures, which is expected to result in diverging Tajima’s *D* patterns. The similar patterns of genome-wide Tajima’s *D* likely also mean that genetic drift has not yet greatly impacted ancestral signatures of selection in these genomes.

Our gene ontology analysis explored the molecular and biological functions, and protein classes associated with genes found in low Tajima’s *D* regions that also harbored significant or fixed SNPs. These included molecular functions associated with binding, catalytic, and nucleic acid binding transcription factor activity, biological functions including metabolic and cellular processes, localization and biological regulation, and protein classes such as enzyme modulators, nucleic acid binding, transcription factors, and transferases, among others (Table S4, Table S5, and Table S6). Future analyses of the functions of these genes might be able to reveal a link to their biological significance in *An. melas*.

Since early studies of host preference, parasitemia rate, and ecology of *An. melas* (Gelfand 1955), and the original taxonomic, genetic, and descriptive studies of the *An. gambiae* complex (Davidson 1962; White 1974), *An. melas* has been considered a malaria vector of minor importance due to its limited distribution and broad host preference. However, early studies focused on populations representing *An. melas* West alone. Recent studies have shown that on Bioko Island, Equatorial Guinea, *An. melas* populations readily feed on humans both indoors and outdoors (Redd *et al.* 2011), and are responsible for up to 130 malaria infectious bites/person/year in the village of Arena Blanca (Overgaard *et al.* 2012). These studies highlight the important role that *An. melas* plays in malaria transmission. The results of this study, in combination with previous work (Deitz *et al.* 2012), indicate that *An. melas* is undergoing an allopatric divergence process. Therefore, what we know about the ecology and behavior of *An. melas* West populations, which have been the focus of the handful of studies on the species (Bryan 1983; Bryan *et al.* 1987; Bogh *et al.* 2007; Caputo *et al.* 2008), may not hold true for the other *An. melas* forms. Additionally, as a member of a species complex that serves as a model for the speciation process, a better understanding of the population genomics of *An. melas* populations enhances our view of how the evolution of the *An. gambiae* species complex is influenced by the diverse host preferences, ecologies, distributions, and demographic histories of its member species.

## Supplementary Material

Supplemental Material
