# Supplementary material for: Genome-Wide Divergence in the West-African Malaria Vector Anopheles melas
Source: G3 (Bethesda). 2016 Jul 27;6(9):2867–79. doi: 10.1534/g3.116.031906 (PMC5015944; doi:10.1534/g3.116.031906)
Supplement: Supplemental Material [file supp_6_9_2867__index.html]

Genome-Wide Divergence in the West-African Malaria Vector Anopheles melas — Supplemental Material 

# Genome-Wide Divergence in the West-African Malaria Vector *Anopheles melas*

## Supplemental Material for Deitz, *et al*, 2016

**Files in this Data Supplement:**

- Table S1 - Results of sequence read trimming, mapping, and filtering. (.pdf, 116 KB)
- Table S2 - *An. melas* population pair-wise, SNP *FST* values per chromosome arm. (.pdf, 95 KB)
- Table S3 - Gene Ontology: Molecular functions for genes harboring significant SNPs found in the bottom 5% Tajima's D regions for the respective populations. (.pdf, 88 KB)
- Table S4 - Gene Ontology: Biological processes for genes harboring significant SNPs found in the bottom 5% Tajima's D regions for the respective populations. (.pdf, 89 KB)
- Table S5 - Gene Ontology: Protein classes for genes harboring significant SNPs found in the bottom 5% Tajima's D regions for the respective populations. (.pdf, 94 KB)
- Table S6 - Mean Patterson's *D*-statistic values per chromosome arm, resulting from the ABBA-BABA test for introgression using the *An. melas* population tree ((West,Bioko)South)*An. gambiae*). (.pdf, 87 KB)
