# Supplementary material for: Genome-Wide Divergence in the West-African Malaria Vector Anopheles melas
Source: G3 (Bethesda). 2016 Jul 27;6(9):2867–79. doi: 10.1534/g3.116.031906 (PMC5015944; doi:10.1534/g3.116.031906)
Supplement: Supplemental Material [file supp_g3.116.031906_TableS1_.pdf]

Table S1 Results of sequence read trimming, mapping, and filtering. Raw sequence reads were trimmed using Trimmomatic ver. 0.35. Paired-end reads were trimmed to a minimum Phred quality score of 20, and a minimum length of 50 bp. Only reads with both surviving pairs were retained for mapping. Reads were mapped and filtered to exclude those with mapping quality values (MAPQ) less than 20.

	Population	**West**				
	SRA Run Accession	SRR567657				
	Number Read Pairs	78,025,712				
	Read Pairs Post Trimming	71,441,506				
	Chromosome	X	2R	2L	3R	3L
	Reference Size (bp)	24,393,108	61,545,105	49,364,325	53,200,684	41,963,435
Raw Reads	Reads Mapped	14,738,106	25,737,921	20,480,713	20,907,372	16,558,122
	Mean Read Length (bp)	98.79	98.88	98.94	99.00	99.04
	Read Length SD (bp)	7.09	6.92	6.83	6.73	6.64
	Mean Reads / bp	57.69	40.54	40.17	38.09	38.17
	Reads/bp SD	1,600.83	250.59	102.95	60.78	573.04
Filtered Reads	Reads Mapped	9,270,784	22,656,116	17,723,120	18,286,869	13,696,759
	Mean Read Length (bp)	98.70	98.88	98.92	99.00	99.01
	Read Length SD (bp)	7.17	6.90	6.83	6.70	6.67
	Mean Reads / bp	36.56	35.76	34.85	33.39	31.65
	Reads/bp SD	814.74	177.19	28.70	28.11	160.39

	Population	**South**				
	SRA Run Accession	SRR567658				
	Number Read Pairs	52,594,743				
	Read Pairs Post Trimming	38,797,029				
	Chromosome	X	2R	2L	3R	3L
	Reference Size (bp)	24,393,108	61,545,105	49,364,325	53,200,684	41,963,435
Raw Reads	Reads Mapped	8,063,847	12,799,679	10,236,490	10,365,790	8,216,090
	Mean Read Length (bp)	98.89	98.96	99.03	99.08	99.11
	Read Length SD (bp)	6.92	6.78	6.65	6.58	6.50
	Mean Reads / bp	31.38	20.04	19.94	18.77	18.84

	Reads/bp SD	977.96	178.37	156.58	32.51	319.12
Filtered Reads	Reads Mapped	4,995,277	11,231,431	8,746,514	9,039,990	6,802,292
	Mean Read Length (bp)	98.84	98.96	99.01	99.08	99.10
	Read Length SD (bp)	6.91	6.74	6.66	6.55	6.50
	Mean Reads / bp	19.58	17.62	17.10	16.41	15.63
	Reads/bp SD	593.91	133.18	16.44	15.62	123.40

	Population	**Bioko**				
	SRA Run Accession	SRR606147				
	Number Read Pairs	56,776,632				
	Read Pairs Post Trimming	50,934,546				
	Chromosome	X	2R	2L	3R	3L
	Reference Size (bp)	24,393,108	61,545,105	49,364,325	53,200,684	41,963,435
Raw Reads	Reads Mapped	11,841,657	18,933,558	15,112,849	15,353,319	12,229,559
	Mean Read Length (bp)	98.80	98.87	98.93	98.99	99.03
	Read Length SD (bp)	7.05	6.92	6.83	6.73	6.64
	Mean Reads / bp	46.32	29.88	29.69	28.03	28.25
	Reads/bp SD	1534.00	234.71	138.21	47.87	362.08
Filtered Reads	Reads Mapped	7,007,348	16,527,538	12,965,972	13,326,759	10,065,430
	Mean Read Length (bp)	98.71	98.86	98.90	98.98	99.00
	Read Length SD (bp)	7.13	6.92	6.85	6.71	6.67
	Mean Reads / bp	27.66	26.13	25.55	24.39	23.31
	Reads/bp SD	723.69	145.95	21.54	19.60	108.90
