# Supplementary material for: Genome-Wide Divergence in the West-African Malaria Vector Anopheles melas
Source: G3 (Bethesda). 2016 Jul 27;6(9):2867–79. doi: 10.1534/g3.116.031906 (PMC5015944; doi:10.1534/g3.116.031906)
Supplement: Supplemental Material [file supp_g3.116.031906_TableS2.pdf]

Table S2 *An. melas* population pair-wise, SNP F_{ST} values per chromosome arm. Regions of heterochromatin in the *An. gambiae* genome were removed from summary statistics.

	X				2R				2L			
Comparison	Q1	Median	Mean	Q3	Q1	Median	Mean	Q3	Q1	Median	Mean	Q3
West - South	0.019	0.032	0.103	0.055	0.021	0.034	0.070	0.060	0.022	0.034	0.074	0.063
West - Bioko	0.013	0.025	0.074	0.043	0.016	0.028	0.075	0.053	0.016	0.029	0.078	0.058
South - Bioko	0.017	0.029	0.147	0.064	0.018	0.033	0.105	0.086	0.019	0.034	0.115	0.099

	3R				3L				Genome-wide			
Comparison	Q1	Median	Mean	Q3	Q1	Median	Mean	Q3	Q1	Median	Mean	Q3
West - South	0.022	0.034	0.072	0.062	0.021	0.034	0.078	0.064	0.021	0.034	0.075	0.062
West - Bioko	0.016	0.029	0.076	0.059	0.016	0.029	0.076	0.057	0.016	0.028	0.076	0.055
South - Bioko	0.018	0.034	0.111	0.092	0.018	0.034	0.116	0.094	0.018	0.033	0.114	0.091
