# Supplementary material for: Genome-Wide Divergence in the West-African Malaria Vector Anopheles melas
Source: G3 (Bethesda). 2016 Jul 27;6(9):2867–79. doi: 10.1534/g3.116.031906 (PMC5015944; doi:10.1534/g3.116.031906)
Supplement: Supplemental Material [file supp_g3.116.031906_TableS3.pdf]

Table S3 Gene Ontology: Molecular functions for genes harboring significant SNPs found in the bottom 5% Tajima's D regions for the respective populations.

Molecular Function Category	West - South	West - Bioko	South - Bioko
antioxidant activity (GO:0016209)	0	1	0
binding (GO:0005488)	13	11	32
catalytic activity (GO:0003824)	20	21	38
enzyme regulator activity (GO:0030234)	3	1	6
nucleic acid binding transcription factor activity (GO:0001071)	6	4	8
protein binding transcription factor activity (GO:0000988)	0	0	1
receptor activity (GO:0004872)	4	5	15
structural molecule activity (GO:0005198)	1	0	9
translation regulator activity (GO:0045182)	0	0	2
transporter activity (GO:0005215)	3	1	9
Total Molecular Function Gene Ontology Hits	50	44	120
Genes	64	62	127
SNPs	95	79	188
