# Supplementary material for: Genome-Wide Divergence in the West-African Malaria Vector Anopheles melas
Source: G3 (Bethesda). 2016 Jul 27;6(9):2867–79. doi: 10.1534/g3.116.031906 (PMC5015944; doi:10.1534/g3.116.031906)
Supplement: Supplemental Material [file supp_g3.116.031906_TableS4.pdf]

**Table S4** Gene Ontology: Biological processes for genes harboring significant SNPs found in the bottom 5% Tajima's D regions for the respective populations.

| Biological Process Category | West - South | West - Bioko | South - Bioko |
|---|---|---|---|
| apoptotic process (GO:0006915) | 2 | 2 | 2 |
| biological adhesion (GO:0022610) | 1 | 2 | 2 |
| biological regulation (GO:0065007) | 8 | 6 | 17 |
| cellular component organization or biogenesis (GO:0071840) | 2 | 1 | 4 |
| cellular process (GO:0009987) | 13 | 11 | 29 |
| developmental process (GO:0032502) | 5 | 5 | 10 |
| immune system process (GO:0002376) | 1 | 2 | 3 |
| localization (GO:0051179) | 8 | 6 | 22 |
| metabolic process (GO:0008152) | 30 | 28 | 55 |
| multicellular organismal process (GO:0032501) | 1 | 2 | 8 |
| reproduction (GO:0000003) | 1 | 1 | 2 |
| response to stimulus (GO:0050896) | 2 | 0 | 7 |
| Total Biological Process Gene Ontology Hits | 74 | 66 | 161 |
| Genes | 64 | 62 | 127 |
| SNPs | 95 | 79 | 188 |
