# Supplementary material for: Genome-Wide Divergence in the West-African Malaria Vector Anopheles melas
Source: G3 (Bethesda). 2016 Jul 27;6(9):2867–79. doi: 10.1534/g3.116.031906 (PMC5015944; doi:10.1534/g3.116.031906)
Supplement: Supplemental Material [file supp_g3.116.031906_TableS5.pdf]

Table S5 Gene Ontology: Protein classes for genes harboring significant SNPs found in the bottom 5% Tajima's D regions for the respective populations.

Protein Class Category	West - South	West - Bioko	South - Bioko
calcium-binding protein (PC00060)	1	0	3
cell adhesion molecule (PC00069)	1	1	0
cell junction protein (PC00070)	0	0	1
cytoskeletal protein (PC00085)	1	0	9
defense/immunity protein (PC00090)	1	2	1
enzyme modulator (PC00095)	2	2	8
extracellular matrix protein (PC00102)	3	2	5
hydrolase (PC00121)	11	10	20
isomerase (PC00135)	0	0	2
kinase (PC00137)	0	1	0
ligase (PC00142)	3	2	4
lyase (PC00144)	0	1	0
membrane traffic protein (PC00150)	0	0	1
nucleic acid binding (PC00171)	7	6	13
oxidoreductase (PC00176)	1	4	4
phosphatase (PC00181)	2	2	2
protease (PC00190)	7	7	17
receptor (PC00197)	4	5	15
signaling molecule (PC00207)	1	1	6
transcription factor (PC00218)	6	4	9
transfer/carrier protein (PC00219)	1	0	2
transferase (PC00220)	1	4	5
transporter (PC00227)	2	1	8
Total Protein Class Gene Ontology Hits	55	55	135
Genes	64	62	127
SNPs	95	79	188
