# Supplementary material for: Genome-Wide Divergence in the West-African Malaria Vector Anopheles melas
Source: G3 (Bethesda). 2016 Jul 27;6(9):2867–79. doi: 10.1534/g3.116.031906 (PMC5015944; doi:10.1534/g3.116.031906)
Supplement: Supplemental Material [file supp_g3.116.031906_TableS6.pdf]

Table S6 Mean Patterson's *D*-statistic values per chromosome arm, resulting from the ABBA-BABA test for introgression using the *An. melas* population tree ((West,Bioko)South)*An. gambiae*).

Chromosome	*D*-Statistic Mean	*D*-Statistic Std. Error	*D*-Statistic Jackknife Mean	*D*-Statistic Jackknife Std. Error	*D*-Statistic Jackknife Z-Score
X	0.030	0.0048	0.030	0.0048	6.15
2R	0.049	0.0029	0.049	0.0029	16.56
2L	0.021	0.0058	0.021	0.0058	3.69
3R	0.045	0.0030	0.045	0.0030	15.10
3L	0.048	0.0035	0.048	0.0035	13.69
Genome-wide	0.040	0.0018	0.040	0.0018	21.80
